# Desmoid tumor-associated pain is dependent on mast cell expression of cyclooxygenase-2

**DOI:** 10.1186/1746-1596-9-14

**Published:** 2014-01-20

**Authors:** Makoto Emori, Mitsunori Kaya, Tomoko Mitsuhashi, Hiroko Asanuma, Toshihiko Yamashita

**Affiliations:** 1 Department of Orthopedic Surgery, Sapporo Medical University School of Medicine, West 16, South 1, Chuo- ku, Sapporo 060-8543, Japan; 2 Department of Surgical Pathology, Hokkaido University Hospital, West 5, North 14, Kita-ku, Sapporo 060-8648, Japan; 3 Department of Surgical Pathology, Sapporo Medical University School of Medicine, West 16, South 1, Chuo- ku, Sapporo 060-8543, Japan

**Keywords:** Desmoid tumor, Cycloxygenese-2, Mast cell

## Abstract

**Background:**

This study aimed to investigate the expression profile of cyclooxygenase-2 (COX-2) in desmoid tumor specimens and to evaluate the correlation of intratumoral COX-2 expression with pain status.

**Methods:**

Sixteen patients with histologically proven desmoid tumors who attended our institution between 2003 and 2010 were enrolled in this study. COX-2 protein expression in desmoid tumors was determined by immunohistochemistry. COX-2 - positive cells had similar morphology to that of mast cells, and therefore, immunohistochemical staining for tryptase was performed in co-localized sections. The number of COX-2 -positive cells in 10 consecutive fields was counted at 400× magnification. Patients were stratified into 2 groups according to the number of COX-2- positive cells, the COX-2 -positive group (≧10 COX-2 -positive cells) and the COX-2 -negative group (<10 COX-2 -positive cells). The prevalence of painful tumors was compared between the 2 groups.

**Results:**

COX-2 was expressed in 9 patients (56%). COX-2 proteins were expressed not in tumor cells but in tryptase-positive mast cells in the stroma of desmoid tumors. 6 of 9 patients in COX-2 -positive group had painful tumors. This difference was statistically significant according to the chi-squared test (*p* = 0 .036), suggesting a positive correlation between COX-2 expression and tumor-associated pain.

**Conclusions:**

Our results indicated that COX-2 secretion from mast cells modulates desmoid tumor-associated pain. In addition, mast cells may at least in part contribute to the pathogenesis of desmoid tumors.

**Virtual slide:**

The virtual slide(s) for this article can be found here: http://www.diagnosticpathology.diagnomx.eu/vs/1490389349103056.

## Background

Desmoid tumors are rare clonal fibroblastic proliferations that arise in the extremities, girdles, chest, abdominal wall, and neck. They are characterized by infiltrative growth and a high risk of local recurrence even after complete surgical excision [1]. Several reports have suggested a link between cyclooxygenase-2 (COX-2) expression and the proliferation of desmoid tumors [2,3]. These findings provide clinical evidence for the use of COX-2 inhibitors in the treatment of desmoid tumors. Most patients present with an asymptomatic, firm, poorly circumscribed mass, but some complain of severe tumor-associated pain. These painful masses often cause decreased joint mobility and functional impairment. COX-2 converts arachidonic acid into prostaglandins, the major modulator of pain. In this study, the hypothesis that COX-2 regulates desmoid tumor-associated pain was examined by immunohistochemical evaluation of COX-2 expression in tumor samples. The relationship between intratumoral COX-2 expression and pain status was evaluated.

## Materials and methods

For the use of these clinical materials for research purposes, prior consent from the patients and approval from the Institutional Review Board of Sapporo Medical University Hospital was obtained.

### Subjects

The study included 16 patients who were treated and followed up at our hospital between April 2003 and May 2010. Needle and/-or open biopsies were performed to confirm the diagnosis of desmoid tumor. Clinical details and follow-up information were obtained by reviewing the patients’ medical charts. All information on tumor-related pain was recorded at presentation.

### Immunohistochemical analysis

COX-2 protein expression in desmoid tumors was determined by immunohistochemistry. Immunohistochemical analysis was performed using the labeled streptavidin-biotin method and tissue sections from paraffin blocks. Immunophenotyping for COX-2 and tryptase was performed using monoclonal mouse primary antibodies for COX-2 (BD Biosciences, San Jose, CA; 1:500) and tryptase (Dako Corp., Glostrup, Denmark; 1:200). Mast cells were identified by tryptase staining. Several previously characterized chondrosarcoma samples were used as positive controls. The immunohistochemical results were evaluated by a pathologist (MT) who was blinded to the patient’s clinical status. The number of COX-2 positive cells in 10 consecutive fields was counted at 400× magnification. The patients were stratified into 2 groups according to the number of COX-2 -positive cells, the COX-2 positive group (≧10 COX-2 -positive cells) and the COX-2 negative group (<10 COX-2 -positive cells)

### Statistical analysis

The chi-squared test was used to examine the correlation between COX-2 expression in desmoid tumors and pain. Statistical significance was defined as *p < 0.05.* Data were analyzed using IBM SPSS Statistics (IBM Corp., Armonk, NY).

## Results

### Clinical characteristics

Details of the clinical features are reported in Table 1. The patients were 2 men and 14 women, 16–80 years of age (median, 36 years). The tumors were located in the back (n = 5), abdominal wall (n = 2), leg (n = 3) [thigh, 2; lower leg, 1], arm (n = 1), axilla (n = 1), and anterior chest wall (n = 1). Treatment procedures consisted were wide resection (n = 4), resection biopsy (n = 2), and simple observation (n = 10). Seven patients (case 2, 3, 7, 10, 11, 12, and 15) complained of tumor pain.

**Table 1:** Details of the clinical features

**Case**	**Age (yrs)**	**Sex**	**Location**	**Pain**	**Duration of symptoms (Month)**	**Clinical characteristics**	**COX-2 expression**	**Treatment**
1	28	F	Thoracic wall (back)	-	-	-	-	WR
2	35	F	Anterior chest wall	+	5	Spontaneous pain, pressure pain	+	WS
3	16	F	Thigh	+	12	Pressure pain	+	WR
4	46	M	Thoracic wall (back)	-	-	-	-	RB
5	33	F	Thoracic wall (back)	-	-	-	+	WR
6	52	F	Knee	-	-	-	-	WS
7	80	M	Abdominal wall	+	3	Pressure pain	+	WS
8	37	F	Abdominal wall	-	-	-	+	WR
9	66	F	Thoracic wall (back)	-	-	-	-	WS
10	33	F	Arm	+	1	Spontaneous pain, pressure pain	+	RB
11	14	F	Calf	+	7	Pressure pain	+	WS
12	67	F	Axilla	+	1	Spontaneous pain, pressure pain	+	WS
13	50	F	Thoracic wall (back)	-	-	-	-	WS
14	25	F	Thigh	-	-	-	-	WS
15	27	F	Thoracic wall	+	1	Pressure pain	-	WS
16	55	F	Thoracic wall	-	-	-	+	WS

M; male, F; female, WR; wide resection; WS, watch and see; RB; resection biopsy.

### Immunohistochemical analysis

**Figure 1:**
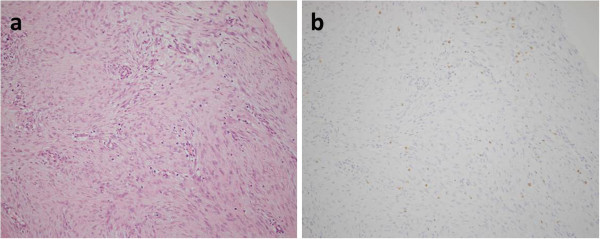
Immunohistochemistry demonstrated no positive staining with COX-2 protein within the tumor cells (X100, (a) Hematoxylin and eosin and (b) COX-2).

**Figure 2:**
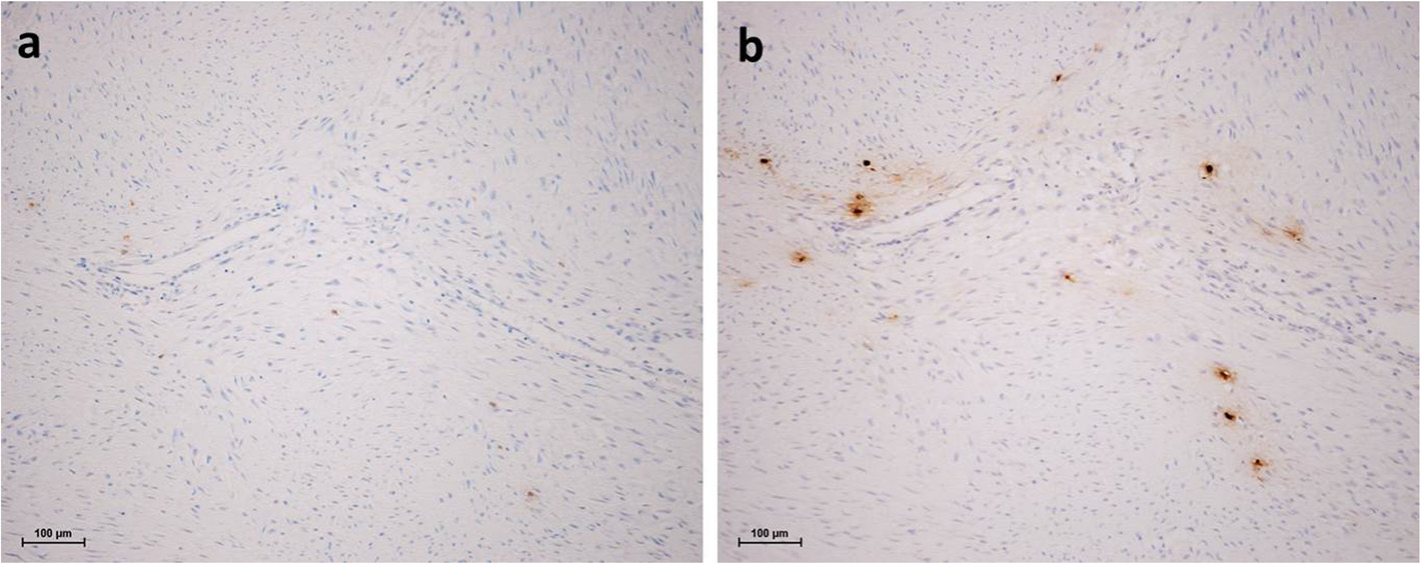
Tryptase clearly co-expressed in the COX-2 positive cells (X100, (a) COX-2 and (b) tryptase).

Immunohistochemistry demonstrated no positive staining for the COX-2 protein within the tumor cells of all samples from the 16 patients (Figure 1). In contrast, several small, round COX-2 -positive cells were detected within the tumoral stroma (Figure 1). COX-2 -positive cells were similar in morphology to mast cells. Therefore, we performed immunohistochemical staining for tryptase in co-localized sections. As shown in Figure 2, tryptase staining was seemed coincided in the COX-2 -positive cells, suggesting that COX-2 proteins were expressed in tryptase-positive mast cells inside the desmoid tumors, and not in the tumor cells themselves. Six out of 7 patients in the COX-2 -negative group had painless tumors. In contrast, 6 out of 9 patients in COX-2- positive group had painful tumors. This difference was statistically significant as assessed by the chi-squared test (Table 2, *p* = 0.036), suggesting a positive correlation between COX-2 expression and tumor-associated pain.

**Table 2:** COX-2 expression and tumor-related pain

	**Painful**	**Painless**
COX-2 -positive group	6	3
COX-2 -negative group	1	6
		p = 0 .036

## Discussion

Mast cells are mobile cells derived from bone marrow that circulate in the blood. Mature mast cells are scattered throughout tissues, including connective tissue [4]. The role of mast cells in connective tissue is still a matter of speculation, and it has been suggested that these cells participate in cell regulation and control of the accumulation of connective tissue components. Several reports indicate that mast cells are potentially fibrogenic, since they secrete potent mediators of fibrosis [5,6]. One of the main components of the secretory granules of mast cells is tryptase [7], an enzyme found exclusively in these cells. Tryptase is involved in diverse biological activities such as fibrogenesis and stimulation of fibroblast proliferation [8,9]. These findings suggest that mast cells are involved in the pathogenesis of desmoid tumors through the action of tryptase, a mast cell-derived protease. Recent studies have shown that tryptase induces COX-2 expression by the specific cleavage of protease-activated receptor-2 [10,11]. COX-2 expression is significantly elevated in several neoplasms, including desmoids tumor [2,3]. COX-2 overexpression leads to increased prostaglandin E2 production, which in turn promotes the growth of desmoid tumors. Furthermore, treatment with COX-2 inhibitors induces the shrinkage of desmoid tumors.

Recent reports have demonstrated that desmoids tumor cells express COX-2 protein [2,3]. However, we failed to show the expression of COX-2 protein within the tumor cells. Conversely, COX-2 -positive mast cells were identified within the desmoid tumors. In addition, a positive correlation between COX-2 expression and tumor-related pain was observed, suggesting that COX-2 secretion from mast cells, not tumor cells, may modulate desmoid tumor-related pain. In addition, there could be connection between mast cells, at least in part, and the pathogenesis of desmoids tumors.

In conclusion, these results suggest a link between clinical symptoms and the tumor microenvironment in desmoid tumors via the secretion of COX-2 from mast cells.

## Abbreviations

COX-2: Cyclooxygenase-2.

## Competing interests

The authors declare that they have no competing interests.

## Authors’ contributions

ME participated in the histological review and drafted the manuscript; MK conceived the study, participated in the histological review, and drafted the manuscript; TM participated in the histological review; HA performed the immunohistochemical study; TY drafted and reviewed the manuscript. All authors have read and approved the final manuscript.

## References

[B1] Penna C Tiret E Parc R Operation and abdominal desmoids tumlrs in familial adenomatous polyposis Surg Gynecol Obstet 1993 177 263 268 8395084

[B2] Signoroni S Frattini M Negri T Pastore E Tamborini E Casieri P Orsenigo M Riva LD Radice P Sala P Gronchi A Bertario L Pierotti MA Pilotti A Cycloxygenase-2 and platelet-derived growth factor receptors as potential target in treating aggressive fibromatosis Clin Cancer Res 2007 13 5034 5040 10.1158/1078-0432.CCR-07-0336 17785554

[B3] Nishida Y Tsukushi S Shido Y Wasa J Ishiguro N Successful treatment with meloxicam, a cycloxygenase-2 inhibitor, of patients with extra-abdominal desmoids tumors: a pilot study J Clin Oncol 2010 28 e107 e109 10.1200/JCO.2009.25.5950 20026797

[B4] Farahani SS Navabazam A Ashkevari FS Comparison of mast cells count in oral reactive lesions Pathol Res Pract 2010 206 151 155 10.1016/j.prp.2009.10.006 20096508

[B5] Garbuzenko E Levi-Schaffer F Emonard H Garbuzenko E Gillery P Maquart FX Activation of fibroblasts in collagen lattices by mast cell extract: a model of bibrosis Clin Exp Allergy 2000 30 485 492 10.1046/j.1365-2222.2000.00737.x 10718845

[B6] Garbuzenko E Nagler A Pickholtz D Gillery P Reich R Maquart FX Human mast cells stimulate fibroblast proliferation, collage synthesis and lattice contraction: a direct role for mast cells in skin fibrosis Clin Exp Allergy 2002 32 237 246 10.1046/j.1365-2222.2002.01293.x 11929488

[B7] Batista AG Rodini CO Lara VS Quantification of mast cells in different stages of human periodontal disease Oral Dis 2005 11 249 254 10.1111/j.1601-0825.2005.01113.x 15984957

[B8] Kondo S Kagami S Kido H Strutz F Muller GA Kuroda Y Role of mast cell tryptase in renal intestinal fibrosis J Am Soc Nephrol 2001 12 1668 1676 11461939 10.1681/ASN.V1281668

[B9] Shimizu Y Suga T Maeno T Tsukagoshi H Kawata T Role of mast cell tryptase-, xhymase + cells in human CD34 bone marrow progenitors Clin Exp Allery 2004 34 1719 1724 10.1111/j.1365-2222.2004.02105.x 15544596

[B10] Wullgus TA Koki AT Zweifel BS Kusewitt DF Rubal PA Oberyszyn TM Inhibition of cutaneous ultraviolet light B-mediated inflammation and tumor formation with topical celecoxib treatment Mol Carcinog 2003 38 49 58 10.1002/mc.10141 14502644

[B11] Frungieri MB Weidinger S Meineke V Kohn FM Mayerhofer A Proliferative action of mast-cell tryptase is mediated by PAR2, COX2, prostaglandins, and PPARgamma: possible relevance to human fibrotic disorders Proc Natl Acad Sci U S A 2002 99 15072 15077 10.1073/pnas.232422999 12397176 PMC137545

